# Materia Medica and Therapeutics; Medical Chemistry; Toxicology and Legal Medicine

**Published:** 1860-01

**Authors:** Emil Fischer


### MATERIA MEDICA AND THERAPEUTICS; MEDICAL CHEMISTRY; TOXICOLOGY AND LEGAL MEDICINE.

BY EMIL FISCHER, M.D.

### MATERIA MEDICA AND THERAPEUTICS.

I.—*Comparative Researches on Oleum Morrhuæ, Rajæ, and Squali.* By M. DELATTRE.

In order to test the statement of M. Homolle, that the oils of the codfish, ray, and shark are of nearly the same composition, M. Delattre has subjected these oils to a careful examination. He prepared them from the fresh livers of these fishes, excluding atmospheric air as completly as possible during the process, and used for this purpose an apparatus of his own invention, consisting of a very large sand bath, in which large balloons of glass are buried to their middle. The livers, cleaned from all impurities, are put into these balloons, and the latter are brought in connection with a reservoir, out of which a stream of carbonic acid is generated, which expels the atmospheric air. This being done, the sand bath is heated to 50° or 60°, the oleum virgineum being gained at 50°, the amber-colored oil at 60°. Especial care is taken to prevent the formation of oleic, sulphuric, or phosphoric acid during the process. The author obtained the following average for the composition of cod-liver oil, (I,) the oil of the ray, (II,) and the oil of the shark, (III,) :—

| | I. | II. | III. |
|---|---|---|---|
| Oleine . . . . . . | 988·70 | 986·94 | 987·17 |
| Margarine . . . . . | 8·06 | 11·01 | 10·12 |
| Chlorine . . . . . | 1·12 | 1·12 | 1·01 |
| Iodine . . . . . . | 0·32 | 0·18 | 0·34 |
| Bromine . . . . . | 0·04 | 0·03 | 0·03 |
| Sulphur . . . . . . | 0·20 | 0·16 | 0·16 |
| Phosphorus . . . . . | 0·20 | 0·28 | 0·20 |
| Loss . . . . . . . | 1·34 | 0·24 | 0·94 |

During his investigations, M. Delattre observed the following important facts: 1. While cod-liver oil contains at common temperature 180 grmm. of margarine in 1000 grmm. of oil, it loses at zero nearly all its margarine, so that it contains not more than 4 grmm. in 1000 grmm.; along with the margarine a corresponding quantity of anorganic substances is separated. 2. Iodine, bromine, chlorine, phosphorus and sulphur do not exist in the oils of these fishes, combined with potassium or sodium, as is generally believed, but in the free state. The three oils, as obtained by M. Delattre, do not, in fact, contain any alkalies at all. 3. During spring the cod-liver oil does not contain any iodine. Further investigations might, therefore, prove the necessity of keeping the oil obtained at that season out of trade.

From experiments made on the sick, M. Delattre arrived at the following conclusions in regard to the therapeutical action of the three oils: 1. The physiological action of all of them is the same; they may, therefore, be used as substitutes for each other. 2. There exists, however, the following difference in regard to their therapeutical applicability: *a.* The cod-liver oil is more efficacious in phthisical patients than the oil of the shark and of the ray. *b.* The oil of the ray is employed with greater advantage in the diarrhœa attending dentition, and in swellings of the mesenteric glands, also in chronic diseases of the skin, and in rheumatism. 3. The oil of the shark is most useful in affections of the bones.

Devergie, who compared cod-liver oil and the oil of the shark with each other, found the latter not only more pleasant, but also quite as efficacious as the former. It is thus an important circumstance, as the supply of sharks (particularly of Squalus catulus) is never deficient, while Gadus Morrhuæ can often not be obtained, and as the shark possesses much less value than the codfish.—(*Bulletin de l'Académie*, xxiv. p. 820, 1859.)

II.—*On different Species of Helleborus.* By PROF. SCHROFF.

After treating in a very thorough manner of the phytography of the different species of helleborus which he has investigated, (Helleborus niger, viridis, orientalis Lam., and fœtidus,) the author proceeds to the second part of his treatise, in which he communicates the results of his experiments with different preparations of these plants. The following is a summary of the most important facts observed by him :—

I. *Results of the experiments made with helleborus niger on rabbits, and on man in health and disease.*—1. The root of helleborus niger, which is inodorous and nearly tasteless, does not possess any volatile active principle, as the fresh root in its totality is just as inefficient as a corresponding quantity of the dried and pulverized root. From one to four and a half drachms of the fresh root acted upon rabbits just as little as one or two drachms of the dried root. 2. The root of helleborus niger possesses but little efficacy; its aqueous and ethereal extracts do not produce any perceptible, or, at the most, but a transient effect. Only the alcoholic extract of the root, obtained in May, is somewhat more active; its administration had a fatal result in two animals, and in a third one it produced very serious symptoms. At all other stages of its development, the root yielded an alcoholic extract which was almost inert. Also the leaves of the plant possess but little activity. 3. The root obtained in May is more active than that gathered at any other period; next to it in efficacy is that obtained in June. 4. Two of the experiments instituted on animals, but particularly the observations made in sick men, prove, in a striking manner, the *cumulative* action of the root of helleborus. Given to rabbits in gradually increased doses, it produced in a few days considerable emaciation, in spite of the existing desire for food, and finally death. In patients to whom the extracts were administered in gradually augmented doses for several days, the first few doses did not seem to have any effect; but, after several doses had been taken, the action of the medicine became perceptible, and increased with each subsequent dose to such a degree of intensity that the medicine had to be discontinued; large quantities were, however, necessary to obtain such a result. Helleborus is thus a medicine to which the organism does not accustom itself, but which, like digitalis, colchicum, aconite, strychnia, and veratria, adds to the action of each subsequent dose that of all the former doses. 5. As in aconite, digitalis, and pulsatilla, the active principles in helleborus are a narcotic and an acrid one. The presence of the narcotic principle is testified by the following symptoms: Heaviness, dullness, stupefaction of the head, vertigo, tinnitus aurium, dilatation of the pupil, sopor, or a restless sleep, interrupted by dreams, reduction of the frequency of the pulse, unusual lassitude, ill humor, anxiety, and apprehension. The presence of the acrid principle, and particularly its action on the digestive apparatus, is proved by the increased secretion of saliva occasionally observed, the vomiting, the pain in the stomach and intestines, the diarrhœa which occurs in exceptional cases, and the increase of the urinary secretion noticed in some instances. Applied to the skin, neither the fresh root of helleborus niger, nor the powder of helleborus orientalis and viridis, made into a poultice with water, produce any irritation, inflammation, or vesication, as is commonly assumed. 6. The immediate cause of death is paralysis of the heart, which is probably owing to the action exerted by the blood upon the ganglionic nervous system. It is remarkable that the irritability of the stomach, small intestines, and heart disappears in an unusually short time, (a few minutes after the last respiration.) 7. The drastic properties ascribed to helleborus have not been confirmed, and the gastro-enteritis, said to follow the exhibition of large doses, has neither been found in acute nor in chronic cases of poisoning. 8. The aqueous extract is inferior in efficacy to the alcoholic extract; the former contains chiefly the narcotic principle, while in the latter also the acrid principle is found.

II. *Experiments with helleborus viridis on rabbits and men.*—1. The root of helleborus viridis, like that of helleborus niger, does not contain any volatile principle. 2. The root as well as the herb possess, compared with that of helleborus niger, a high degree of efficacy. Two drachms of the fresh, or one drachm of the dried and pulverized root, produced death in seven and seven and a half hours, while four and a half drachms of the fresh root, and one to two drachms of the dried root of helleborus niger gave rise to no perceptible symptoms in animals. Doses of fifteen grains of the pulverized root make a decided impression, but do not produce death of the animal. One drachm of a stronger alcoholic extract killed the animal in one hour; the same dose of a weaker extract in three hours. Ten grains of the alcoholic, ethereal, and aqueous extract caused death always in from four to twelve hours, while one or two drachms of the aqueous and ethereal extract of helleborus niger produced but little effect. The extracts of the leaves acted in a corresponding manner. 3. Also helleborus viridis contains a narcotic and an acrid principle, but in much larger quantity than helleborus niger. The symptoms are the same as produced by helleborus niger, but are much more intense; the irritation of the intestinal canal especially is much more violent, and attended with fluid discharges, (even after a dose of two to four grains;) the diuresis is much increased. 4. Helleborus viridis, like helleborus niger, produces death in consequence of the paralysis of the heart. The depression which induces the paralysis is, however, often preceded by a stage of exaltation, during which the action of the heart and the lungs becomes more rapid and tumultuous, and congestion of the blood takes place to the brain and its membranes, and to the spinal marrow, which may give rise to hemorrhages. 5. Also from the use of helleborus viridis a distinct gastro-enteritis has never been observed. 6. The aqueous extract is not inferior in efficacy to the alcoholic extract; the alcoholic extract of the leaves is at least quite as active as the alcoholic extract of the root.

III. *Experiments with helleborus orientalis.*—(The roots with which the experiments were made were obtained from M. Landerer, of Athens.) 1. The root of helleborus orientalis considerably surpasses the root of helleborus niger, and even that of helleborus viridis, in efficacy; it is the true representative of the action of helebori. One drachm of the pulverized root was fatal in four hours; one drachm of the alcoholic extract in twenty-five minutes; one drachm of the aqueous extract in two hours. Five grains of the alcoholic extract produced in rabbits chronic poisoning, which led gradually to a high degree of emaciation, and to peculiar rigid contractions of the posterior extremities. 2. Helleborus orientalis contains an acrid and a narcotic principle, but in greater quantity than helleborus viridis. 3. Like helleborus niger and viridis, helleborus orientalis causes death by paralysis of the heart; the preceding stage of excitement is still greater than that observed after the use of helleborus viridis. 4. Gastro-enteritis could not be discovered. 5. The alcoholic extract proved to be more active than the aqueous. One drachm of the former produced death in twenty-five minutes; one drachm of the latter in two hours; ten grains of the alcoholic extract were fatal in less than two hours; the same quantity of the aqueous extract in seven hours. The ethereal extract, which seems to contain particularly the acrid principle, is still to be examined.

IV. *Experiments with helleborus fœtidus in animals.*—As the author could not obtain the fresh plant for his experiments, he does not consider them completed; he is, however, able to state the following facts: 1. Helleborus fœtidus surpasses the helleborus niger in the poisonous properties of its root and leaves, but is, however, inferior to helleborus orientalis, and even to helleborus viridis. One drachm of the alcoholic extract of the leaves of helleborus fœtidus caused death in two hours; one drachm of the alcoholic extract of the fibres of the root in half an hour; one drachm of the whole root in one hour; one drachm of the aqueous extract in six hours; while equal doses of the corresponding extracts of helleborus niger did not produce any, or but a slight effect. 2. The experiments with helleborus fœtidus also prove the presence of a narcotic and of an acrid principle. In what proportion they are present, and whether a third principle is united with them, as the peculiar odor of the plant seems to indicate, are questions to be decided by further investigations. The acrid principle seems to be most readily extracted by ether, and should, therefore, preponderate in the ethereal extract; but the symptoms during life seem to indicate that the narcotic principle predominates; the animals suffered of prostration, of diminished frequency of the pulse, and of respiration, of spasms in various parts of the body, and died either after an attack of convulsions, or gradually under the symptoms of paralysis. 3. Death is caused likewise by paralysis of the heart. 4. The plant and the extracts gradually lose their efficacy even with the most cautious preservation.—(*Prager, Vierteljahrschrift*, xvi. 2, 1859; and *Schmidt's Jahrbücher*, 1859, No. 10.)

III. *On Ranunculus Ficaria.* By STANISLAUS MARTIN. (Bull. de Thérap., lvi., p. 518, 1859.)

The author communicates his chemical investigations on the composition of the dried and fresh leaves, the bulbs and roots of ranunculus ficaria. Without giving the proportions, he asserts having found in the leaves fatty substance, volatile oil, traces of ficarine, chlorophylle, vegetable albumen, vegetable extractive matter, (the last two in the fresh leaves;) in the dried leaves a yellow coloring matter, probably a product of decomposition of the chlorophylle. In the bulbs, he found ficarine, volatile oil, starch, traces of ficaria acid, ligneous fibre; in the fresh root the same substances, but much more of ficarine and ficaria acid. The ficarine is light yellow; at the beginning of a sweetish, then slightly acid and astringent taste; soluble in water and alcohol, with which it foams; it burns on red-hot coals, with a disagreeable odor, unlike that of caramel; assumes with sulphuric acid a rose color; with chloride of iron, with caustic potassa and caustic lime, it does not undergo any change in color; on fermentation it does not form alcohol. The ficarine can be obtained in several ways, but the best process is the following: The peeled root is bruised to the consistency of pap, and extracted, in the balneum Mariae, with double its weight of alcohol of 28°; then often shaken, and, after three days, boiled for fifteen minutes; the liquid is decanted on cooling, the pap pressed out, the liquid filtered, and the alcohol removed by distillation. The residue is then treated with cold water, the solution filtered, and evaporated to the consistence of an extract. If starch is still present the ficarine must be treated with heated alcohol of 30°, filtered, and then the alcohol distilled off. The ficarine is to be kept in tightly-closed vessels.

It is well known that Plinius recommended the plant in hemorrhoids, but since the end of the last century the remedy seems to have been forgotten. The author recommends ficarine as a lotion in hemorrhoids, in the proportion of one part to fifty parts of distilled water; or as an ointment or liniment, in the proportion of one part to thirty parts of fat or glycerin.

V. Holsbeek states, (in the Presse Médicale, 22, 1859,) that the root of ranunculus ficaria, or ficaria ranunculoides, is well known in the country surrounding Brussels as a popular remedy in hemorrhoidal affections. It is used, in the form of powder, half a drachm to one drachm; decoction, one ounce and a half to two ounces to two pints of water; tincture, one part to six of alcohol; extract, one part to six of water; and syrup. In cases complicated with constipation, V. Holsbeek found pills made of the extract, in combination with extract of nux vomica, very useful. He prescribed two grains of the extract of ranunculus ficaria and one-eighth of a grain of extract of nux vomica, to be taken every morning and evening; and ordered at the same time fumigations with the steam of the decoction, and the introduction of a tent into the anus, smeared with an ointment containing extract rad. ficar. ranunc. and opium.

In Germany the efficacy of this plant in hemorrhoidal affections has been recently brought into notice again by Neuhausen.—(*Organ f. d. ges. Heilkunde*, ii. 4; *Schmidt's Jahrbücher*, September, 1859, ix.)

IV. *On Glycerin, and its Applications in Surgery and Medicine.* By M. DEMARQUAY. (Pamphlet, octavo, 42 pp. Labé: Paris, 1859.)

It is about five years since attention was directed to the advantages which the employment of glycerin presented, especially in surgery. M. Demarquay was one of the first to make use of this substance, and it is the result of a long experience which he makes known in his recent treatise.

The first part of it is devoted to the history and pharmacology of this liquid; it is produced, as is well known, in the fabrication of soaps and candles, but often remains impure, and does not present the necessary qualities; it is then more injurious than useful. We must therefore assure ourselves at first of the purity of the liquid which we are going to employ; the best glycerin is known at present by the name of *English glycerin;* it must not form a precipitate with the salts of baryta, sulphuric acid, hydrosulphuret of ammonia, and oxalate of ammonia; the adulteration with glucose is recognized by boiling the liquid with a small piece of caustic potassa, when the adulterated product assumes a dark color. Pure glycerin is neutral, as is proved by litmus-paper.

After having pointed out the conservative properties of glycerin, in regard to organic, and particularly animal substances, M. Demarquay speaks of its application in the dressing of wounds in general; the dressing is much more simple and more proper than that with cerate; the deposit of crusts around the wound is avoided; but particularly in cases of wounds of bad nature the use of glycerin is indicated; this liquid prevents the development of hospital gangrene, and causes the disappearance of this complication when it is developed, on the use of ordinary dressings. It is used with advantage in the dressing of gangrenous wounds, and particularly of those which succeed the incision made in anthrax. In military surgery it is particularly useful, in cases of contused wounds abundantly suppurating, when the dressing cannot be renewed often enough, and when the wounds, in consequence of the accumulation of the wounded, and from bad hygienic conditions, have a tendency to assume an unfavorable character.

In cases of deep abscesses, with burrowing of matter and fistulous passages, glycerin is advantageously employed, in order to check the abundance of suppuration, to clean the secreting surfaces, and to modify the bad quality of the pus; the liquid can be injected into these anfractuous sores; in the same way it may be used in the sinuous passages which are so often formed in stumps after amputation.

M. Demarquay then points out the utility of glycerin in dressing burns, all kinds of ulcers, chancres, chaps, and chilblains. In diseases of the external ear and affections of the eyes glycerin is equally serviceable.

We have not yet finished with the applications which can be made of this substance; it may perhaps be believed that the properties of so inert a liquid as glycerin are exaggerated; M. Demarquay protests against the prejudice which considers all those substances inert and without real action which are inoffensive and require no great precaution or prudence in their management; yet glycerin, already very useful in itself, can be rendered still more active by the addition of some other medicines, such as laudanum, tannin, iodine, aloes, etc.

A combination of glycerin with tannic acid, (*glycerole de tannin,*) for instance, is used with advantage in the treatment of vaginitis; the preparation is composed as follows:—

| | |
|---|---|
| Glycerin . . . . . . | 100 parts. |
| Tannic acid . . . . | 10 to 20 " |

"The speculum having been introduced, an injection of water is applied, in order to remove all the purulent mucus adhering to the walls of the vagina; these, afterwards, are wiped with a dossil of dry charpie placed at the end of a long forceps. I introduce then one or more tampons of cotton, soaked in the tannic glycerin; and, in addition to it, a dry tampon, destined to retain the drops which tend to escape."

It may be useful to add a little tannin to the glycerin employed for dressing wounds. The reporter of the Journal du Progrès, who has treated some cases with this application, states that it seems to accelerate cicatrization.

Finally, M. Demarquay terminates his interesting memoir with the use made of glycerin in the treatment of diseases of the skin, and reports some experiments which he has made with glycerin taken internally.—(*Journal du Progrès*, September 30, 1859.)

V. *On Ozonized Oils.* By THEOPH. THOMPSON, M.D., F.R.S. (Read before the London Medical and Chirurgical Society.)

The author, after some general remarks on the properties of ozone, describes the results obtained from its administration in association with oils; the oils being ozonized by exposure for a considerable time to the direct rays of the sun, after previous saturation with oxygen gas, according to the process adopted by Mr. Dugald Campbell. The cases of fourteen consumptive patients to whom the ozonized oils were given are detailed, and the principal facts noted are also appended, in a tabular form. The conclusion to which these experiments point is, that the administration of ozonized oils has a remarkable tendency to reduce the frequency of the pulse. Of the fourteen patients whose cases are detailed in this communication there are only two in whom no such effect was observed; and although in a few instances the effect may have seemed insignificant or transient, in the larger proportion it was very considerable, and must be attributed to the ozone rather than to the oil, since it was repeatedly manifested in patients who had taken cod-liver and other oils without any reduction, or even with an acceleration of the pulse; and further, the effect on the pulse was nearly as distinct when the ozone was associated with the oil of the cocoa-nut or of the sun-flower, as with that of the cod-liver. This circumstance is the more significant since the administration of sun-flower oil without ozone has not appeared to the author to manifest any important remedial power. The reduction of pulse was usually observed in two or three days, and often continued progressive. A reduction of twenty beats was observed in certain cases to occur respectively in two, three, four, and six days; in other instances a reduction was noted of twenty-four pulsations in fourteen days, thirty-four in thirteen, thirty-six in twenty-two, forty in eleven. In one patient the pulse fell as low as sixty, probably considerably below the natural standard; but in most of the favorable instances the reduction stopped when that standard was obtained.

The apparent effect of the remedy is one which, prior to experiment, the author would not have anticipated. No other obvious result was noticed, excepting a general improvement in the patient's condition. In some of the patients the use of simple and of ozonized oils was alternated. In one case the alternation was made three times, and the result was in each interchange of treatment so direct and remarkable as to make that particular example equivalent in force to three experiments. In addition to the patients under his own observation, the author refers to four instances noted by Dr. Scott Alison, who obligingly pursued the investigation during Dr. Thompson's absence from the hospital. In these four cases the disease was in the third stage. In two, a remarkable reduction in the rapidity of the pulse, amounting to about twenty beats, occurred under the use of the ozonized oil, while the improvement induced could not be referred to any other cause.

Dr. Alison remarks: "I attach some value to this observation; for I prescribed the oil totally divested of all prejudice in its favor, and I have always been reluctant, on imperfect grounds, to refer results to the operation of medicines. If ozonized oil can reduce the rapidity of the circulation—a feature of great prominence in phthisis—this remedy possesses a most valuable property, rendered still more valuable by its contributing at the same time to improve the general health."

The author mentions having used ozonized oil of turpentine with marked and prompt advantage in some cases of hæmoptysis, but has not sufficiently repeated the experiment to feel entitled to express an opinion as to its remedial superiority over ordinary turpentine. He adds that, should more extended observation establish for ozonized oil the property indicated by these experiments, it will prove a valuable addition to our list of remedies, especially in consumption, which is a disease peculiarly characterized by hurried action; but not, perhaps, exclusively in this disorder, since there are other morbid conditions in the treatment of which it is very important to lower the pulse without reducing constitutional strength.—(*Dublin Med. Press.*, vol. xlii. p. 35.)

VI. *A Substitute for Fowler's Solution.* By DR. TH. CLEMENS.

Dissatisfied with Fowler's solution, which is liable to decomposition, and frequently adulterated with sulphate of lime, etc., the author has used for a long time the arsenite of bromide of potassium, (arsenigsaures Bromkali,) for the preparation of which he gives the following formula:—

℞.—Acid. arsenios. pur.,
Potass. carbonat. pur., āā ʒj;
Coq. cum Aq. dest., ℔ss.
Ad perfect. Solut., refrig.; adde
Aqu. dest. q. s. ut fiat solut., ℥xij.
Dein adde Brominii puri, ʒij.

This solution, which must be shaken during the first week several times daily, is, in four weeks, colorless, and then ready for use; it is to be kept well stopped, and protected from the light. It excels by its rapid, not destructive, but tonic effect upon the system. The preparation suggested itself to the author, from the fact that many mineral waters contain arsenious acid and bromine, to the presence of which, he believes, they owe principally their efficacy. He prescribes it in doses of three to four drops, to be taken in a glass of water one or two times daily, and has thus given it for many years without noticing any injurious effects. He has administered it with excellent results in intermittent fever, different chronic cutaneous diseases, secondary and tertiary syphilitic diseases of the skin, secondary syphilitic swelling of the testicle, and in white swelling of the knee. The author reports a great number of cases which serve to support his statements.—(*Deutsche Klinik*, 1859, 10–12.)

VII. *Creosote in Cough with Purulent Expectoration.* By PROF. HIRSCH.

Prof. Hirsch recommends, in his "Clinical Fragments," ii., the use of creosote in cough with purulent expectoration. He states that he discovered its efficacy by chance, in treating a case of protracted pneumonia, attended with profuse purulent sputa, emaciation, and hectic fever, which made him suspect the existence of tubercular infiltration, undergoing rapid softening; he prescribed creosote as a palliative against the troublesome vomiting from which the patient suffered, and found, to his astonishment, that not only the vomiting but also the cough and expectoration rapidly disappeared. Since that time he has administered creosote in doses of half a drop to one drop, in pills, in many cases where these symptoms existed, and has found it very efficacious.—(*Foriep's Notizen aus dem Gebiete der Natur und Heilkunde*, 1859, iii., 20.)

VIII. *Muriate of Gold and Belladonna in Affections of the Stomach and Chronic Vomiting.* By PROF. J. HOPPE.

Prof. Hoppe relates the case of a woman, thirty-two years of age, of rather weak constitution, who had suffered for nearly six years from vomiting of a thin, watery, sometimes acid fluid, and from a gnawing pain and feeling of emptiness in the stomach; the author prescribed one grain of muriate of gold in one ounce of water—ten drops of this solution to be taken four times daily, in a cup of water. All the unpleasant symptoms disappeared completely, and the appetite of the patient was considerably increased. After some time the gnawing sensation returned, and the patient was put on the use of extract of belladonna, of which she took one grain within two weeks, with the effect of obtaining perfect and permanent relief.—(*Preussiche Vereins-Zeitung*, N. F., ii., 24, 1859.)

IX. *Chloroform in Hooping-cough.* By DR. R. FLEHINGER.

Dr. Flehinger states, that by the use of chloroform in hooping-cough he has not only shortened the duration of the disease, but also the length of the paroxysms. The paroxysms do not occur so often, do not last so long, and the children are less exhausted and debilitated by them. The cough frequently ceases within six to eight weeks. The author uses chloroform in combination with olive oil, in a liniment composed of equal parts of both, which he orders to be rubbed on the breast, the sides, and the back; and, if there is much vomiting, also in the region of the stomach. If the paroxysms are very violent, and return frequently, Dr. Flehinger orders frictions with chloroform, without olive oil; and if the disease is complicated with bronchitis he recommends inhalation, which, however, is not to be carried so far as to produce anæsthesia. For this purpose he uses fifteen to twenty drops, on cotton, every two hours, continuing the application from several seconds to two minutes. He found it easy to carry out this mode of treatment even in the youngest patients; the result is often surprising, and chloroform is, according to the author's experience, the only remedy by which we are at present able to alleviate this disease.—(*Memorabilien aus der Praxis*, 30, 1859.)

X. *Infusion of the Leaves of Rhamnus Alaternus as an Antigalactic.*

The editor of the "Floriligio Medico di Roma" reports several cases of lying-in women, who, from different reasons, could not put their children to the breast, or had to cease nursing them, and used the infusion of the leaves of rhamnus alaternus for the purpose of stopping the secretion of milk. In all of them the secretion was quickly diminished, the turgid state of the breasts rapidly reduced, and the patients thus saved the trouble arising from this condition, and the inflammation so often following it. He adds, that he found it impossible to discover the least disturbance in the other secretions and excretions of the women to whom he had administered the remedy. He is therefore inclined to believe that the rhamnus alaternus exercises a specific action upon the mammary glands; and he proposes to apply this remedy also to other swellings of these glands. The rhamnus alaternus is an evergreen shrub, which is cultivated in gardens as an ornament, and is known in Italy by the name of "Olivelle," on account of the configuration of its leaves. It is given in infusion, the dose being five to six leaves to two pints of boiling water.—(*Zeitschrift für Wundärzte*, etc., 1859, xii. 1.)

XI. *Digitaline in Puerperal Fever.* By Dr. Serre.

In one of the late sessions of the Académie de Médecine of Paris, Dr. Serre communicated an elaborate treatise on the treatment of child-bed fever, in which he extols the efficacy of digitaline in this affection. He administered one granule of the preparation "digitaline," manufactured by Homolle and Quevenne, every four, five, or six hours. He reports nine cases treated in this manner, in eight of which the remedy acted very favorably, although the disease had made already considerable progress in some of them. The principal effect of it consists in diminution of the frequency of the pulse and of respiration, and a simultaneous amelioration of all the other symptoms. The efficacy of digitaline is, according to his statement, superior to that of quinine, which has been recently recommended, and produces a similar effect.—(*Gazette des Hôpit.*, 1859, 50.)

XII. *Arsenious Acid in Chorea.* By M. E. Barthez.

M. Barthez gives a detailed account of the case of a girl, eight years old, who had been quite healthy previously, but, in consequence of a sudden fright, had been attacked with chorea, six weeks before coming under his care. The patient recovered in a week, under the use of about one-twelfth to one-sixth of a grain of arsenious acid three times daily.—(*Gazette des Hôpit.*, 68, 1859.)

XIII. *Hœmatic Capsules.* By M. Foy.

M. Foy, a talented pharmacien, of Paris, proposes to give to chlorotic, weak, or convalescent patients, capsules containing extract made from the blood of the calf, sheep, or ox. The preparation of these capsules is extremely simple; no desiccation, trituration, or pulverization is required. The blood is simply to be evaporated in vacuo, and to the extract a certain quantity of phosphate of soda is to be added, to assist the gastro-intestinal solubility of the solidified fibrin. The proportions are, extract of arterial blood of calf, one pound; phosphate of soda, thirteen drachms; mix thoroughly, and make capsules of from five to ten grains. Each capsule contains a small quantity of iron, this very minute amount insuring the absorption of the metal, and assimilating the hæmatic capsules to the natural chalybeate waters. From ten to twenty capsules a day may be given, beginning with those made with the blood of the calf.—(*London Lancet*, December, 1859.)

XIV.—*Glycerin Ointment for the Itch.* By M. Bourguignon.

M. Bourguignon, so well known in Paris by his successful researches on "the acarus scabiei," has published, in the Gazette Médicale, the following formula. One general friction, not preceded by soap ablutions, is sufficient: Yolks of two eggs; essence of lavender, lemon, and mint, of each seventy-five drops; essence of cloves and cinnamon, of each 120 drops; gum tragacanth, half a drachm; well-pounded sulphur, twenty-six drachms; glycerin, thirty-two drachms. Total weight, nearly eleven ounces. Mix the essences with the yolks of eggs, add the gum tragacanth, make a good mucilage, and then add very gradually the glycerin and sulphur.

Many cures have been obtained by this preparation, which has the advantage of giving no pain.

The well-known Helmerich ointment, being really useful, M. Bourguignon has modified it, and substituted glycerin for the lard. In the altered form the preparation is not any dearer, as efficacious, and less painful than the original ointment. It does not grease the cloths, and has an agreeable perfume. Gum tragacanth, fifteen grains; carbonate of potash, thirteen drachms; well-pounded sulphur, twenty-six drachms; glycerin, fifty-two drachms; essence of lavender, lemon, mint, cloves, and cinnamon, of each fifteen drops. Total weight, nearly eleven ounces. Make a mucilage with the gum and one ounce of glycerin, add the carbonate, mix until it is dissolved, and then gradually add the sulphur and glycerin; lastly, pour in the essences. With this compound, M. Bourguignon advises two general frictions of half an hour, within twelve hours of each other, and followed, twenty-four hours afterwards, by a simple warm bath, as the glycerin is soluble in water. Two-thirds of the preparation should be used for the first friction, and the other third for the second.—(*London Lancet*, December, 1859.)

XV. *Bromine in Pseudo-membranous Affections.* By DR. CH. OZANAM.

To the cases already reported by him, Dr. Ozanam adds six others, four of membranous angina of the fauces, (one with scarlet fever,) and two of tracheal croup, in which the bromine water, prepared by him, has effected a cure within a few days.

It is worthy of notice, that chloroform destroys the odor of bromine so completely that it is only necessary to open a bottle of chloroform in order to make the odor in a room filled with vapor of bromine immediately disappear.

This effect is owing to the formation of bromoform, the odor of which is much less than that of bromine.—(*Gazette des Hôpit*, 72, 1859.)

### MEDICAL CHEMISTRY.

I. *On the Tests for Detecting Sugar in the Urine.* By DR. WIEDERHOLD, of Göttingen. (Pamphlet, 8vo., 2d ed. Göttingen: J. H. Wiegand, 1859.)

The examination of urine, with a view to ascertain the presence of sugar in it, is almost daily required from the practitioner. The comprehensive treatise written by Dr. Wiederhold is well calculated to aid the physician in his task. The summary of it consists in the following:—

The cases in which the presence of sugar in the urine is to be ascertained are of two kinds.

1. The quantity of the urine secreted during twenty-four hours exceeds by far the normal average, (2000 CC.,) and its specific gravity is, at the same time, abnormally high, (more than 1020.) The urine has a greenish-yellow color, and tastes and smells sweetish sour. In these cases it is sufficient to apply Trommer's test directly, and it will be rarely the case that the diagnosis of diabetes mellitus cannot be positively proved by this means. To render it still more sure, the fermentation test may be used, and sugar in substance prepared from the urine.

2. The urine does not possess the qualities described above; but there are reasons to suspect a beginning diabetes, or the presence of sugar is to be ascertained merely out of scientific interest, either in a physiological or pathological point of view; the following procedure is, then, the best to adopt: As large a quantity as possible of the urine is evaporated to dryness on the water bath, and the residue extracted by treating it repeatedly with alcohol. One part of the alcoholic solution is again evaporated on the water bath, and the residue formed by the evaporation dissolved in distilled water. This solution is then subjected to Trommer's test or to the silver test. If the experiments do not prove decisive, a solution of hydrate of potassa, in absolute alcohol, is added to the other part of the alcoholic solution. After some time, when the precipitate formed by the admixture has settled down on the walls of the glass, the supernatant clear liquid is poured off, and the residue washed several times with anhydrous alcohol. This being done, the residue is dissolved in water, and the aqueous solution is filtered with finely pulverized (chemically pure) charcoal prepared from blood, until the filtered liquid runs off clear and colorless. This solution, which, if necessary, may be somewhat concentrated by evaporating it on the water bath, is then tested for sugar, with an alcoholic solution of copper, or by means of nitrate of silver. If the reduction to the red suboxide or the formation of a silver film takes place, the presence of sugar is positively proved. In the contrary case, the absence of sugar can be, according to the author's experience, positively assumed.—(*Foriep's Notizen*, iv. 6, 1859.)

II. *On the Green or Blue Color of Pus.* By M. C. BERGOUHNIOUX.

The author is of the opinion that, with the use of some dexterity, the blue or rather green color of pus can be easily proved to be owing to the coloring matter of the bile. The bandages and dressings, soaked with pus, are washed in water, alcohol, or ether, and the pigment is obtained in as pure a state as possible. This being done, some bile—not fresh, however, but such the color of which resembles that of the dressings—is treated in the same manner as the liquid obtained by the washing of the dressings, and thus a solution similar to that derived from the pus is obtained. If both fluids are treated with the reagents generally employed in testing for biliary pigment, exactly the same phenomena will be observed in both solutions.

The author states that he has proved in this manner the presence of biliary pigment in meconium, in diarrhœal discharges, in the liquor amnii, in vomited matter, in the pus of urethritis, otitis, ophthalmia, coryza, arthritis, syphilitic or scrofulous abscesses of the glands, in serous exudations, in hydropic fluids, cadaveric infiltration, and many other normal and pathological fluids.

The blue or green color of the pus has been ascribed to widely different substances. Now, it was said to be owing to a favus, then to a cyanogen compound, to sulphuret of iron, etc. It is possible that various substances impart the peculiar color to pus. It is, however, worthy of notice, that Dr. Schiff found ferrocyanuret of iron in the coloring matter obtained in a mechanical manner from blue pus, a substance which has been found already several times in other parts of the organism.—(*Gazette de Paris*, i., 1859.)

### III. *Researches on the Sugar found in the Glucogenous Matter of the Liver.* By MM. Berthelot and S. De Luca.

It is known from the experiments of M. Cl. Bernard, that the glucogenous matter of the liver can be transformed into a particular glucose. But the nature of this glucose, and its specific characters, have not yet been precisely determined. We do not know, for instance, whether this glucose is identical with some of the different kinds of glucose known at present, such as glucose of grapes, of malt, lactic glucose, etc., or whether the hepatic glucose is a new kind, endowed with peculiar properties.

Having succeeded in obtaining the hepatic glucose,* in combination with chloride of sodium, in the crystallized form, we subjected this definite compound to a systematic examination.

It presents itself under the form of voluminous, limpid, colorless crystals, capable of reducing the tartrate of copper and potassa, and of fermenting under the influence of yeast.

These crystals are rhombohedrons of 78 degrees. Their rotatory power, determined by the aid of an aqueous solution, is directed toward the right; it was found to be equal to $+47^{\circ}$. This power is much more considerable in the first moments which attend the solution of the crystals.

These crystals, finally, contain 8·3 of chlorine, which corresponds with the formula,

$$2C^{12}H^{12}O^{12}, 2HO + Na\,Cl.$$

All these properties agree exactly with those of the combination of grape sugar with chloride of sodium, as known by the investigations of M. Peligot and M. Pasteur.

Thus the identity is proved of the glucose formed by means of the glucogenous matter of the liver and the ordinary glucose, that is to say, of the glucose of grapes and of diabetes.—(*Journal de Pharmacie et de Chimie*, October, 1859.)

### Toxicology and Legal Medicine.

### I. *Blood-Crystals, and their Judicial Medical Importance.* By L. Buchner and G. Simon.

Büchner and Simon, in a valuable paper on blood-crystals, give as characteristic of these the subjoined particulars: They usually form rhomboidal tables, but seldom rhomboidal columns. When somewhat imperfect, they take the form of a shuttle. Their color changes from yellow to black, but most frequently they are of a dirty-red brown. Their size is various; they generally lie in groups together, and often lie crossways, form the X figure, or they take the stellate

---

* Formed by the reaction of diluted hydrochloric acid on the glucogenous matter of the liver of the rabbit.

form. Forms of needles, rods, or grains, are of little value in a forensic point of view. The crystals are insoluble in water, alcohol, acetic acid, phosphoric acid, and hydrochloric acid. They dissolve with difficulty in ammonia, in dilute sulphuric acid, and in dilute nitric acid; but are easily soluble in potash water, English sulphuric acid, and in the vapor of nitric acid. In chlorine water they lose their color, and look as if corroded. For the demonstration of the crystals, a very small quantity of blood, or of fluids tinged with blood, is required, but an excess of concentrated acetic acid is necessary. The character, age, or impurity of blood, in no way hinders the crystallization. To obtain crystals, a drop of fluid blood is to be mixed with a small excess of concentrated acetic acid, and evaporation is slowly made over a spirit-lamp; in the dried matter remaining the crystals will be recognized by the microscope. In cases where the blood which has to be examined has been robbed of its salts by washing, rain, or moisture, as may happen, crystallization will not occur by the simple process given above. Under these conditions it is necessary to add a few grains of common salt, when the capability of crystallization is restored. It is, perhaps, after all, best in forensic inquiries to use the salt on every occasion. A source of fallacy may enter inquiries of this nature, for it has been found that madder, red ink, dragon's blood, and some other substances, will also form crystals. Crystals thus formed may be distinguished by their want of color, irregular form, solubility in water, and different chemical reactions. Muroxide offers the greatest difficulty in determination, since it forms crystals similar to hæmatin crystals, both in color and form. Solution of muroxide gives, however, with acetic acid, a residue after evaporation of a clear brick-red color. This residue, covered with water, dissolves, giving a purple-red color. Addition of hydrochloric acid destroys the color, and potash causes dissolution with a blue color. Blood-crystals, on the other hand, are insoluble in the first, and are dissolved in potash with a green color.—(*Virchow's Archiv*, Band xv.; *British and Foreign Medico-Chirurgical Review*, October, 1859.)

### II. *Case of Rape committed on a Child.*

Dr. D. McKinlay relates the particulars of a case of rape committed by an adult on the person of a child of less than seven years of age. The injuries produced on the person of the child were remarkably extensive. They are thus described by Dr. McKinlay: "At the upper part of the cleft of her buttocks, behind and above the anus, the skin was besmeared with dried blood. In her private parts the vagina was lacerated in various directions. One laceration extended from the lower part of the vagina downward, dividing the recto-vaginal septum and perineum, on the left side of the raphé, down to the verge of the anus, and laying bare the rectum. Another laceration, having divided the upper part of the vagina, passed upward along the left nympha, and terminated near the root of the clitoris. The vagina was also lacerated laterally, so that for an inch and three-quarters inward there appeared an open lacerated cavity, or pouch, rather than the semblance of a continuous canal.

"In the cavity produced by the laceration there was some feculent matter. This was found to have come through a lacerated opening in the coats of the rectum. Three-fourths of an inch inward from the verge of the anus there was a lacerated opening in the rectum of nearly an inch in length, through which the fæces escaped into the lacerated cavity before described. This opening had evidently been produced at the same time with the other parts of the laceration, and was not owing to any subsequent sloughing of the parts."

On the clothes of both the accused and the little girl stains were found, in which, by the microscope, blood-globules, epithelial scales, and spermatozoa were detected. In the course of the treatment of the case it was found necessary to divide that portion of the recto-vaginal septum which extended between the verge of the anus and the laceration. It appears that the child gradually recovered, although for some time the action of the sphincter was not established.—(*Glasgow Medical Journal*, July, 1859, p. 135.)

III. *On the Physiological and Toxicological Properties of Tanghinia Venenifera.* By PROF. PELIKAN.

This tree grows in Madagascar, and belongs to the family of apogineæ, (to which also vinca and nerium oleander belong;) it contains a milky juice; its most poisonous part is the fruit, a stone-berry, similar to a lemon, with a stone resembling that of a peach, which is the principal seat of the poison. Prof. Pelikan had an alcoholic extract prepared from the leaves and stalks of the plant, and, aided by Prof. Kölliker, experimented with it on frogs. The experiments proved that it does not belong to the tetanic poisons. Its effect is particularly directed upon the heart, the action of which it paralyzes, leaving the ventricles in a bloodless condition; this effect is a direct one, and not brought about merely by the medulla oblongata and the spinal marrow. Secondarily, it paralyzes the motor nerves in the direction from the centre toward the periphery; tertiarily it paralyzes the muscles of voluntary motion. The tanghinia is thus to be considered a specific poison for the heart and the muscles; which, however, paralyzes the muscles less rapidly than upas antiar, veratrine, and sulpho-cyanide of potassium, but which, in regard to its paralyzing action upon the heart, is nearly equal to antiar, and surpasses considerably the two other poisons.—(*Verhandlungen d. Phys.-Med. Gesellschaft in Würzburg*, ix. i.; *Foriep's Notizen*, 1859, iv. 6.)

IV. *On the Effect of Inhalation of Arseniuretted Hydrogen.* By M. GOSEBRUCH.

According to M. Gosebruch, the inhalation of arseniuretted hydrogen, like that of hydruret of antimony, produces its poisonous effects in consequence of the poisoned blood not taking up oxygen any more, and of the hæmatoglobuline passing into a state of solution.—(*Foriep's Notizen*, 1859, iv. 5.)

